# Comparison and Impact of Different Fiber Debond Techniques on Fiber Reinforced Flexible Composites

**DOI:** 10.3390/polym12020472

**Published:** 2020-02-18

**Authors:** Julia Beter, Bernd Schrittesser, Boris Maroh, Essi Sarlin, Peter Filipp Fuchs, Gerald Pinter

**Affiliations:** 1 Polymer Competence Center Leoben GmbH, Roseggerstrasse 12, 8700 Leoben, Austria; Bernd.Schrittesser@pccl.at (B.S.); Boris.Maroh@pccl.at (B.M.); PeterFilipp.Fuchs@pccl.at (P.F.F.); 2 Faculty of Engineering and Natural Science, Tampere University, P.O. Box 589, 33014 Tampere, Finland; essi.sarin@tuni.fi; 3 Department of Polymer Engineering and Science, Montanuniversitaet Leoben, Otto Glöckelstrasse 2, 8700 Leoben, Austria; gerald.pinter@unileoben.ac.at

**Keywords:** flexible composite, fiber-matrix adhesion, interface, bundle pull-out test, fiber-matrix debond technique

## Abstract

The focus of this paper is the realization and verification of a modified fiber bundle pull-out test setup to estimate the adhesion properties between threads and elastic matrix materials with a more realistic failure mode than single fiber debond techniques. This testing device including a modified specimen holder provides the basis for an adequate estimation of the interlaminar adhesion of fiber bundles including the opportunity of a faster, easier, and more economic handling compared to single fiber tests. The verification was done with the single-fiber and microbond test. Overall, the modified test setup showed the typical pull-out behavior, and the relative comparability between different test scales is given.

## 1. Introduction

Fiber reinforced elastomers (or so-called flexible composites) represent a new class of materials. These composites connect the targeted improvement of the mechanical properties due to the reinforcing fibers used and still retain sufficient flexibility provided by the elastomer matrix. Completely new applications for the technical advances could be found, e.g. in the field of medical engineering to generate artificial muscles [1], exoskeletons for rehabilitation [2], or self-folding structures [3] where flexibility is essential and stability must be guaranteed to provide sufficient mechanical performance. The focus has been on the possibility of load transfer between fibers and their surrounding matrix due to an external input such as a mechanical trigger. Thus, good adhesion properties are important to ensure an efficient load transfer [4], which provides a reduction of the stress concentration as well as an improvement of the general mechanical properties themselves [5]. In-depth knowledge as well as quantitative investigation of the interface properties are essential [6] since an excellent fiber-matrix bonding is important [7]. Currently, the individual approaches regarding the experimental solutions have been limited to the analysis of single fiber-matrix interactions, and, moreover, have usually been reduced to specifically selected material groups like carbon or glass fiber with epoxy thermoset systems. The comparability between various existing test setups is only possible within a limited range. Therefore, an adequate estimation of the material behavior in component-like structures containing a large number of fiber bundles involves numerous boundary conditions. Thus, a direct correlation with the macroscopic composite parts is not feasible [4,6]. Currently, there are several methods to investigate the interface properties in a single fiber and fiber bundle scale [7,8,9,10]. Generally, the single fiber push-out [11] and the pull-out tests [12,13,14] or the fiber bundle pull-out (FBPO) test [15,16,17,18] represent a fiber-loaded system. In these tests, the common measuring tests are carried out for a clamped matrix and therefore less suitable for elastic materials [19]. In contrast to proven test methods at the single fiber scale, the already existing fiber bundle test methods do not show consistent or systematic design. For example, in some systems, the fiber bundle is fully coated with matrix material [4,16], while the matrix block is fixed, which might influence the mechanical behavior [20]. Another setup describes a process in which the fiber bundle sample is extracted from an already produced composite [21]. Existing FBPO tests do not consider a guiding element to eliminate bending [8], which is important for elastomers. In terms of the various test setups, the FBPO test offers the opportunity to perform experiments faster, more easily, and more economically compared to single fiber measuring devices, which need more careful handling [22] and special test equipment [4]. Besides that, the tests are influenced by the following factors: e.g., (i) the complex stress distribution in a fiber bundle [13,23], (ii) fiber-fiber interaction [24] with more real failure modes [23], or (iii) the presence of statistically distributed filaments inside the fiber bundle [25,26]. These points have a significant effect on the interlaminar shear strength due to the variable cross-section areas of the fiber bundle [4]. Nevertheless, single fiber measuring methods have a high experimental outlay [22], and the determination of parameters for the fiber-matrix bonding behavior is only possible when assuming specific material models. 

The aim of this research is to present the first development of a modified fiber bundle pull-out test to characterize the adhesion properties of a fiber bundle in elastic matrix. With this test setup, clamping influences or other effects like coated fibers are avoided, which ensures an easy, fast, and economic handling to estimate the fiber-matrix adhesion with more real failure modes. A comparison of the presented test setup with proven test methods based on single fibers was performed [7]. One focus was the verification of the FBPO test with the purpose of a mechanical test standardization as a method to estimate the damage behavior of a fiber bundle when being pulled out of the surrounding matrix. The influence of several parameters was analyzed, namely embedded length, speed, and adhesion properties due to different fiber-matrix combinations. The described modified FBPO test setup aims to provide the basis for an adequate estimation of the properties between a fiber bundle and the surrounding matrix material including more realistic failure modes (e.g., statistical fiber distribution or fiber-fiber interaction). Further, the obtained material data can be used in constitutive approaches for elastic body simulations [27]. In this work, the possibility to obtain the common characteristic parameter maximum force was proven. In addition to that, the adaptation to design a suitable sample manufacturing device including a modified specimen holder was carried out. Additional experiments were performed with proven measuring devices on single fiber scale via microbond and single fiber pull-out (SFPO) tests.

## 2. Materials and Methods 

### 2.1. Materials

For the experimental investigation of two different fiber types, glass fibers (GF), and polyester fibers (PETF) were chosen as reinforcement. The commercial unidirectional GF reinforcement was provided by CS Interglas AG (Erbach, Germany) as E-type, an area weight of 220 g/m^2^ ± 5%, a twine thickness of 68 tex, and a mean fiber diameter of about 10 µm with the standardized warp yarn classification EC9-68x5t0 in the 0° and 90° direction. The PETF reinforcement was provided by Mates Italiana srl (Milan, Italy) from a single batch and with a 2/2 twill weave, an area weight of 200 g/m^2^ ± 5%, a twine thickness of 167 tex, and a mean fiber diameter of about 30 µm with an area bundle distribution of 50/50 in the 0° and 90° directions. Extracted from the unidirectional weave, the GF bundles possessed different surface conditions: (a) with commercial silane-based surface treatment, as received from the supplier (finish FK144 treated), and (b) without surface treatment obtained by cleaning the fibers with peroxymonosulfuric acid (untreated). The cleaning procedure was essential to achieve an unmodified status of the GF material. This process was performed at 60 °C for 120 min, including a thorough washing step with toluol and a drying step at 150 °C for at least 12 h in a convection oven.

Two different cast elastomers, polydimethylsiloxane (PDMS) and polyurethane (PUR) were considered as elastic matrix material. According to the manufacturer, the PDMS (Elastosil RT601 provided from Wacker Chemie AG) is a two-component-system rubber used in the mixing ratio 9:1 (part A: part B), and has a density of 1.02 g/cm³ and a viscosity in the uncured mixed state of 3500 mPas including a pot life up to 90 min at 23 °C. The PUR (ClearFlex 50 provided from Smooth On Inc.) with a mixing ratio of 1:2 (part A: part B) has a density of 1.04 g/cm³. The viscosity of the mixed product in uncured state is 250 m Pas with an indicated pot life limited to around 25 min at 23 °C. 

### 2.2. Preparation and Impregnation Quality of Fiber Bundle Specimens

**Figure 1:**
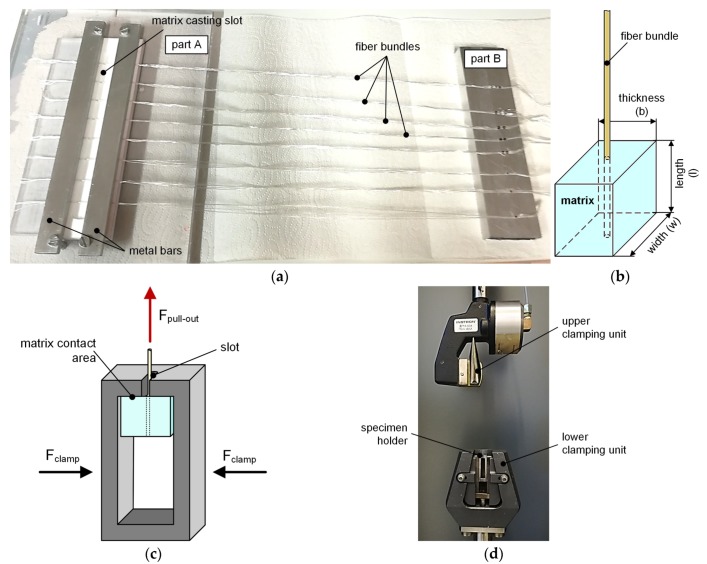
Manufacturing tool including main components (**a**) with specimen (**b**) and schematical test procedure with the modified specimen holder (**c**) with test setup for FBPO test (**d**).

For the preparation of the FBPO test specimens, a tool (see Figure 1a) was developed. This tool consists of a corpus which contains a defined recess for the matrix including narrow slots occurring at regular intervals for the fiber bundles. In addition, regarding the recess, seals were implemented on both sides to guarantee a definite fiber position without pre-damage and to prevent matrix leakage. The issue was the accurate fiber position in the center of the specimen to avoid negative effects related to tilting or asymmetrical stress distribution. Each specimen, shown in Figure 1b, had the same thickness b of 8 mm as well as width w of 10 mm, and the length l was adjusted according to the desired embedded length l_e_.

### 2.3. Specimen Holder

A modified specimen holder was designed to accommodate the cuboid specimen geometry as well as to be able to test the samples with a conventional testing machine. During the test, this holder has to prevent slipping. Also clamping of the surrounding matrix has to be avoided to reduce further stresses induced by the fixing unit, because these stresses were transferred to the elastic matrix and further directly into the embedded fiber bundle, which result in a fiber breakage. A specimen holder [17] originally designed for carbon black filled rubbers was modified for the testing on unfilled elastomer systems. All fiber bundle specimens have to be placed, deformation-free, inside the specimen holder. During the time to reach the preload for equal test conditions, the elastomers were sensitive to small surface imperfections which could lead to tilting or twisting. The specimen holder enables the guidance of the lateral surfaces of the specimen in parallel direction to the lateral inner surfaces of the specimen holder, hereby still providing sufficient space on both sides between the specimen and the holder. This allows the surrounding matrix material to undergo deformation during the loading without causing any additional stresses that might affect the experiment in a negative manner (see Figure 1c).

### 2.4. FBPO Test

The test setup for the fiber pull-out testing represents a combination of the standardized testing method for pure yarn tension testing, according to the ASTM D2256 enhanced with the specimen holder for the modified FBPO test, shown in Figure 1d. This test setup represents the fiber-loaded measuring device, where the upper clamping device fixes the fiber bundles and represents the movable part [28]. The tests were performed with a constant displacement rate v pull out. Generally, all experiments were carried out with a universal testing machine (5500 Series, Instron GmbH) and performed at standard atmosphere conditions. For the test procedure, a 100 N load cell and a defined gauge length of 50 mm was chosen. In order to have the same initial starting condition, all experiments were carried out with a preload of 1 N. As the upper clamping device for the fiber bundles, the mandrel-shaped clamping system was used and the specimen holder was inserted in the lower clamping device. As can be observed from Figure 1c, the matrix part was centered between the inner lateral surfaces and was only in contact with the upper surface area during the testing to avoid lateral clamping forces. 

To evaluate the influence of the test parameters, one material combination (represented by silane-based treated GF with PDMS matrix) was employed representatively. Due to the influence of the pull-out speed v pull out, and embedded length of the fibers mentioned in the literature [13,19], a methodically validated testing plan was developed (see Table 1). The embedded length as well as the pull-out speed were tested at three different levels, including all possible combinations in each case. As the reference setting 5 was chosen.

In order to determine the maximum force for the obtained interface properties, the average value for at least five specimens for each setting was calculated. Besides the influence of the test parameters (i.e., embedded length, pull-out speed), the impact of different fiber-matrix combinations using the same test setup was analyzed in a subsequent step. Therefore, the GF and PETF were combined for each setting with both matrix materials and measured with the same reference setting 5 similar to the first testing plan with an embedded area of 10 mm as well as a pull-out speed of 1 mm/min.

### 2.5. Single Fiber Pull-Out Test

**Figure 2:**
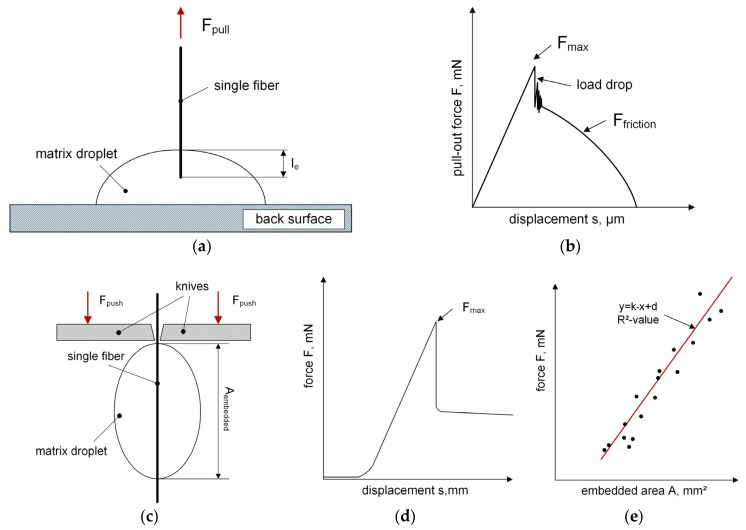
Schematic test procedure (**a**) and a typical force-displacement graph (**b**) of the SFPO test [13,22] with schematical test setup (**c**) and typical test graphs (**d**,**e**) of a microbond test [29,30].

The experiments to investigate the adhesion behavior of one single filament was carried out with the SFPO tester [14] at the Federal Institute for Materials Research and Testing (BAM, Berlin, Germany). These tests were performed to verify the modified test setup used for the FBPO tests as well as to prove the reproducibility for identical fiber-matrix combinations. The SFPO test emphasized a similar loading situation compared to the FBPO test. This test set-up was designed so that the specimen holder containing the matrix droplet was placed in the rigid test device with a fiber orientation perpendicular to the matrix surface. One fiber end was impregnated with the matrix droplet, which also allowed defining the corresponding embedded length individually, as shown in Figure 2a. The fixed end of the single fiber is loaded until a fiber detachment from the surrounding matrix in the interface occurs and is then pulled out completely. The load displacement signal of each specimen was measured to determine the required maximum force until the initiation of the debonding with the indicated load-drop (see Figure 2b). The tests were performed with a 50 N load cell, and a defined pull-out speed of 1 µm/s. All samples were manufactured with an embedded length of about 186 µm using the same specimen preparation conditions as for the FBPO tests.

### 2.6. Microbond Test

The investigation of the fiber-matrix interface properties was carried out with the FIBRObond micro-droplet tester [29,30] at the Tampere University (Tampere, Finland). The reason for using this test setup was to assess the fiber-matrix adhesion properties in different loading conditions, i.e., matrix loading in the microbond test versus fiber loading in the SFPO test, as well as to determine their comparability. With the test procedure shown in Figure 2c, the force that is needed to detach the matrix droplet from the attached filament area is determined. Several matrix droplets with different sizes were applied on the surface of one single fiber. In order to determine the necessary debonding and maximum force, each individual matrix droplet is loaded until the debonding takes place at a constant displacement rate of 0.5 mm/min (see Figure 2d). The maximum debonding force and the corresponding embedded area are reported as one data point in the maximum force-embedded area diagram, as shown in Figure 2e. Due to different matrix droplet sizes, several data points resulted due to the different debonding forces required, and a curve with a linear correlation was evaluated in conclusion. Generally, for each setting, about 15 droplets were tested to ensure sufficient accuracy and determined with the R^2^-value. Hence, the evaluated parameter k represents the slope, and thus, the adhesion property, so that a higher k-value indicates a stronger interlaminar bonding. All tests were carried out with a 1 N load cell at standard atmosphere conditions.

### 2.7. Optical Observation of Fracture Surfaces

To enhance the characterization as well as the comparability of the tested single and fiber bundle tests, optical damage analysis was performed using an optical microscope (BX51, Olympus Austria GesmbH, Vienna, Austria), and the scanning electron microscopy (SEM) (Tescan Vega II, Tescan Brno, s.r.o., Dortmund, Germany). Regarding all FPBO tests, a camera system (Prosilica GT 6600, Allied Vision Technologies GmbH, Stadtroda, Germany) was operated additionally to follow the material behavior during the debonding process more accurately.

## 3. Results and Discussion

### 3.1. Fiber Bundle Pull-Out Test

**Figure 3:**
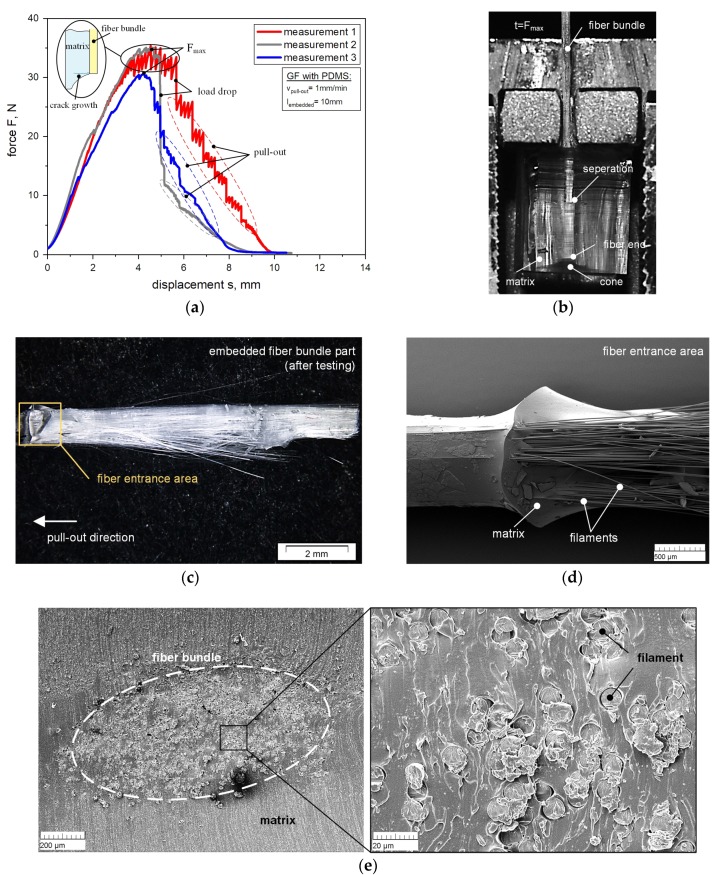
Force-displacement curves obtained from FBPO test on GF with PDMS matrix (**a**) with the separation process (**b**) during pull-out and microscopic images of a FBPO specimen (GF with PDMS) after the test: part of embedded area of GF- bundle (**c**) and SEM of GF- bundle (**d**) and image of cross-section area of embedded GF bundle (**e**).

Concerning the FBPO tests, all experiments show the typical shape of the load-displacement curve for the type of failure occurring at the interface area [14,19,26] and is represented in Figure 3a for three measurements with the reference setting, respectively. A reason for this deviation for the same setting could be differences in the adhesive connection to the fiber, which might be influenced by inhomogeneities of the commercial silane-based surface treatment from the supplier. Other influences could be the presence of interface defects or deviations of fiber bundle orientation due to the placing process. These influences can be reduced by optimizing the manufacturing process of the samples. The load-displacement curve illustrates the increased force during the test until it reaches the maximum force F_max_, and then to the clearly discernible load drop shown in Figure 3a, at which point the fibers separated from the surrounding matrix. After complete debonding, the fiber bundle is pulled-out with F_pull-out_, which contains several different parts including the friction force. Some tests exhibited a stepwise crack growth that led to an oscillation in the load signal, which was optically recorded as a crack initiation perpendicular to the pull-out direction. A reason for this behavior could be several mechanisms for fiber-matrix bonding, such as mechanical interlocking, adsorption interaction, or the enabling of elastic deformation due to oversized free clamping distance of the fiber bundle between both clamping units. Therefore the fiber bundles could probably bear elastic deformation energy, which would be released during the debonding procedure and could probably cause compression stresses which would lead to a vertical stress reduction and crack initiation [6,10,31]. More experiments varying the free clamping distance and other upper fixing devices for the fiber bundles should be carried out in further investigations to optimize the pull-out behavior. During the loading procedure, a cone at the end of the fiber bundle inside the sample is visible, where the elastic matrix is deformed, and drawn in the direction parallel to the fiber bundle (see Figure 3b). In the meantime, the adhesive bonding of the surrounding matrix is undamaged. The separation process can be recognized by the shift of the light refraction. The crack initiation starts on top of the embedded area, where only the matrix surface is in contact with the bottom area of the specimen holder. The reason for this could be the stress concentration of the already deformed elastomer [11]. Due to the elasticity of the matrix, a slight deformation in the pull-out direction was observed since a small gab between the fiber bundle and the lateral surface of the slot has to be considered to avoid friction. Further, no lateral contraction of the fiber bundle during the stretching was depicted. Because of this compression of the elastic matrix, the local matrix surrounding the fiber bundle starts to peel perpendicular to the fiber orientation, and therefore, tension stresses to the fiber bundle, which leads to the separation effect [7,10,13]. Figure 3c represents the optical microscopic images of the embedded area of one GF bundle with the PDMS matrix after the FBPO test. It is visible that the complete GF bundle was pulled out without fiber breakage. The initiation of debonding at the fiber entrance area is shown in more detail in Figure 3d in the SEM picture. The separation happened at the interface area and was initiated by the stress concentration occurring on top of the fiber entrance area into the matrix as well as at the fiber end [6,11]. SEM images were taken from the cross-section area to ensure a complete impregnation of the fiber bundle, which is exemplary shown for GF with PDMS matrix (see Figure 3e).

**Figure 4:**
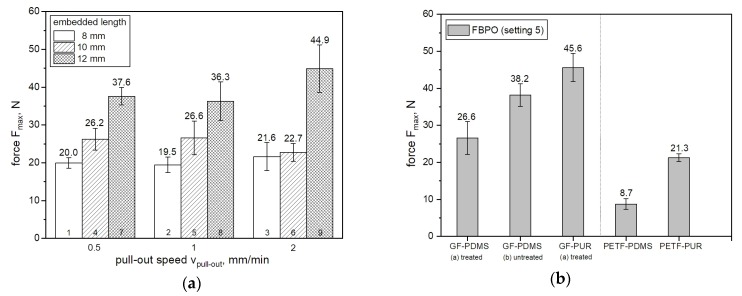
Influence of the parameters on the pull-out force for GF and PDMS matrix (**a**) and on different reinforcement-matrix material combinations tested on reference setting (**b**).

For the determination of the pull-out behavior due to the influence on the test parameters (i.e., embedded length, pull out speed) themselves versus the maximum force F_max_, the results of GF with PDMS matrix are shown in Figure 4a. As expected from previous researches [19,32] focusing on the investigation and impact on the interface behavior as well as debond mechanism influenced by different embedded lengths, the maximum pull-out force increases with higher embedded length. A reason for this is the fact that there is more adhesive bonding due to the increased embedded length (more fiber bundle surface). There is no clear effect on the influence of the pull-out speed regarding the viscoelastic behavior of the matrix material to relate to higher force values. This could be due to the fact that the range of 0.5 to 2 mm/min was too small to achieve sufficient influence on the elastic material. However, the standard deviation tends to increase with higher pull-out speed, such as for setting 1 compared with setting 3. For the investigation to verify the sensitivity of the measuring by the modified test setup for FBPO testing, the results concerning the material influence due to different fiber-matrix combinations is presented in Figure 4b. Generally, the results reveal that the GF-bundles for both matrix materials (PDMS and PUR) lead to a higher maximum force, which may be related to the higher number of single fibers in the fiber bundle and the larger effective surface area between fibers and matrix resulting thereof. The highest pull-out force was obtained with GF(a)- PUR matrix. Moreover, the pull-out force required for PETF with a PUR matrix was higher, with about 21.3 N, than PETF with a PDMS matrix with approximately 8.7 N. However, in terms of the GF bundles, the results show that the removal of the silane-based surface treatment of the GF increased the pull-out force to about 45.6 N. A possible reason for this could be (i) the incompatible sizing provided by the supplier or (ii) the presence of rougher surface conditions [11,25] induced by the piranha acid treaded GF bundles, which was applied to remove the silane-based treatment from the manufacturer. Moreover, all reported measurements for GF show generally higher deviations in contrast to the results with PETF.

As an overview, the results of each fiber-matrix combination for all the different test methods (FBPO, SFPO-, and microbond test) have been summarized in Table 2.

### 3.2. Comparison Between Different Test Scales

**Figure 5:**
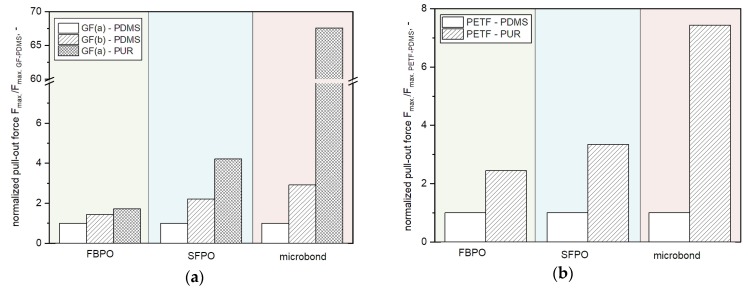
Comparison of the relative pull-out force for FBPO, SFPO and microbond tests performed with different fiber-matrix combinations with GF (**a**) and PETF (**b**).

For a suitable comparison of the FBPO test with other test methods (SFPO- and microbond test), a normalization was carried out on the basis of the two reinforcement fiber types (GF as well as PETF) in combination with the PDMS matrix regarding the maximum force needed to debond the fiber bundles from the matrix, as shown in Figure 5a,b.

Reasons for this are as follows: (i) both fiber types have different properties such as surface quality or fiber diameter, (ii) comparability is needed for tests at different measurement scales (micro-meso level), and (iii) the sensitivity of the measurements should be better visualized. For the normalization of the microbond tests, one pre-defined embedded area was chosen with the corresponding force value.

Generally, the results reveal that for the individual test setups, the same fiber-matrix combinations follow the same trend regarding the corresponding relative pull-out force F_max, normalized_. In terms of measurement sensitivity, it can be seen that the difference for all tests of the FBPO test setup with GF is smaller than in comparison to the measurement methods on single fibers. Thus, the combination GF(a) with the PUR matrix has four times higher F_max, normalized_ than GF(a) with the PDMS matrix, which is the normalized reference setting. The comparatively low measurement sensitivity of FBPO tests can be related to several factors, such as (i) interaction effects between single fibers in the fiber bundle by fiber friction, (ii) the statistical distribution of single fibers and the matrix in the bundle, and (iii) different shear stress distribution upon pull-out.

## 4. Conclusions

The modified FBPO test setup here described is intended to provide the basis for an adequate estimation of interlaminar properties between a fiber bundle and the surrounding matrix material including more realistic failure modes (e.g., statistical fiber distribution or fiber-fiber interaction) with the aim of a mechanical test standardization. The obtained material data can be used in constitutive approaches for elastic body simulations. With the FBPO test, it is feasible to perform the experiments in a faster, easier, and more economical way compared to the single fiber debond technique devices which require a more careful handling.

The results of the FBPO tests reveal that the separation procedure occurred at the interface area. Additional studies for the various fiber surfaces to influence the adhesion with tailored chemical fiber surface modifications as well as matrix material combinations are currently being carried out to prove the measurement sensitivity and to study the interfacial surface interactions. Further researches are already in progress to investigate the other important failure phenomena, e.g., load drop, friction force, and debonding initiation to get a deeper knowledge of the physical phenomena behind the presented test. Another aspect is the analysis of the specimen geometry focusing on the lateral surface dimensions and their effect on the cone during testing. Overall, the results reveal that the modified FBPO test setup with both measurement methods on a single fiber (SFPO- and microbond test) is relatively comparable, including the fact that these test setups belong to different measuring scales. Knowing this, verification of the modified FBPO test is validated, which allows sufficient accuracy of the material pre-analysis for adhesion properties in a faster and easier way. Moreover, additional studies should be carried out regarding the comparability to macro scales for composite structures, and that the presented modified FBPO test method provides a base model as well as a possible link in the test chain between micro scale- (single fiber) and macro scale (composite components) testing.

## Figures and Tables

**Table 1:** Methodical testing plan for FBPO tests.

Setting	1	2	3	4	5	6	7	8	9
embedded length l_e_, mm	8			10			12		
pull-out speed v_pull-out_, mm/min	0.5	1.0	2.0	0.5	1.0	2.0	0.5	1.0	2.0

**Table 2:** Results for FBPO-SPFO and microbond test for different fiber-matrix combinations.

Fiber-Matrix	FBPO	SFPO	Microbond	
	av. max. Force Fmax, (± std. dev.), N	av. max. Force Fmax, (± std. dev.), mN	Slope (k), mN/mm^2^	R^2^ -
GF (a)-PDMS	26.6 ± 4.5	11.6 ± 1.4	0.01	0.8
GF (b)-PDMS	38.2 ± 3.0	25.7 ± 1.8	0.1	0.7
GF (a)-PUR	45.6 ± 3.8	48.8 ± 15.0	1.4	0.8
PETF-PDMS	8.7 ± 1.5	19.5 ± 10.5	0.2	0.8
PETF-PUR	21.3 ± 1.1	65.3 ± 15.0	1.4	0.9
